# Supplementary material for: A Human Brain-Chip for Modeling Brain Pathologies and Screening Blood–Brain Barrier Crossing Therapeutic Strategies
Source: Pharmaceutics. 2024 Oct 10;16(10):1314. doi: 10.3390/pharmaceutics16101314 (PMC11510380; doi:10.3390/pharmaceutics16101314)
Supplement: Supplementary file 1 [file pharmaceutics-16-01314-s001.zip › pharmaceutics-3178416-supplementary.pdf]

pharmaceutics
MDPI

Supplemental Figures

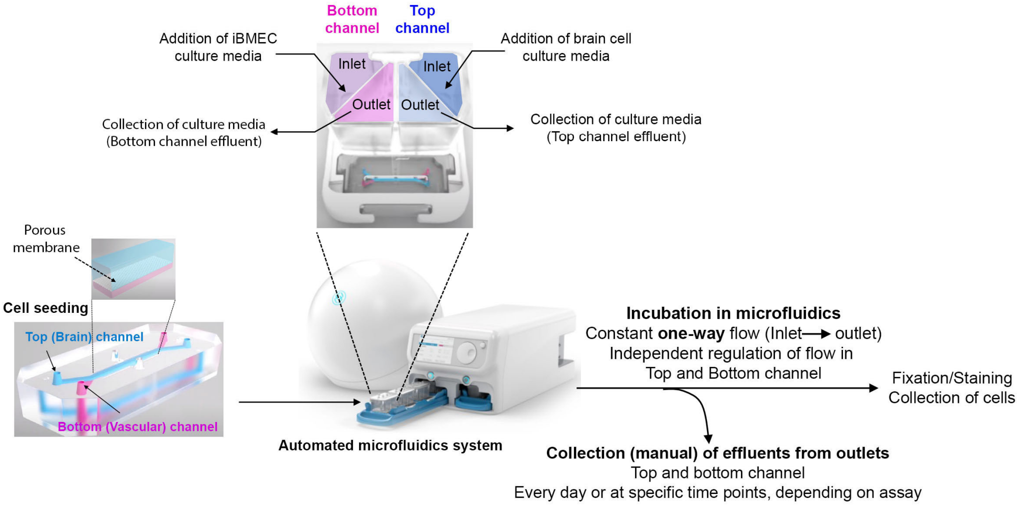

Figure S1. Overview of human Brain-Chip experimental setup. Endothelial cells are incorporated in the bottom (vascular) channel and are separated from the parenchymal compartment (top or brain channel) by a semi-permeable membrane coated with tissue-specific extracellular matrix. After cell seeding, the Chips are connected to the Pods which supply culture media to the top and bottom separately. Fresh culture media are added to the inlet and flow into the channels. Effluents are collected in the outlets. The chips are cultured in the automated microfluidics system which regulates a constant one-way flow in top and bottom channels independently. Effluents are collected at different time points for analysis based on the assays. At the end of each experiment, cells are fixed or collected for downstream analysis.

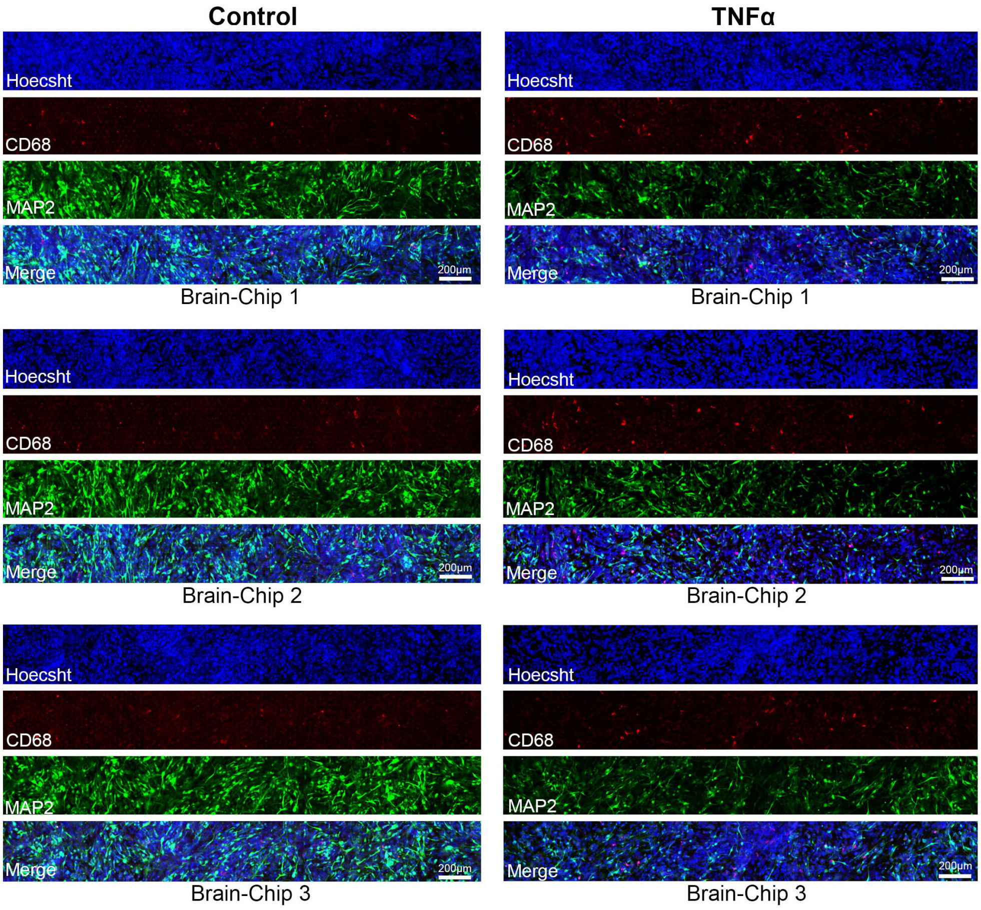

Figure S2. Activation of microglia and neuronal damage in the Brain-Chip upon TNFα treatment. Confocal images (stack of z-series) of microglia (CD68) and neurons (MAP2). Increase of CD68 positive cells and reduction of MAP2 indicate microglial reactivity and neuronal damage in response to TNFα, respectively. Supplementary data for Figure 2B.

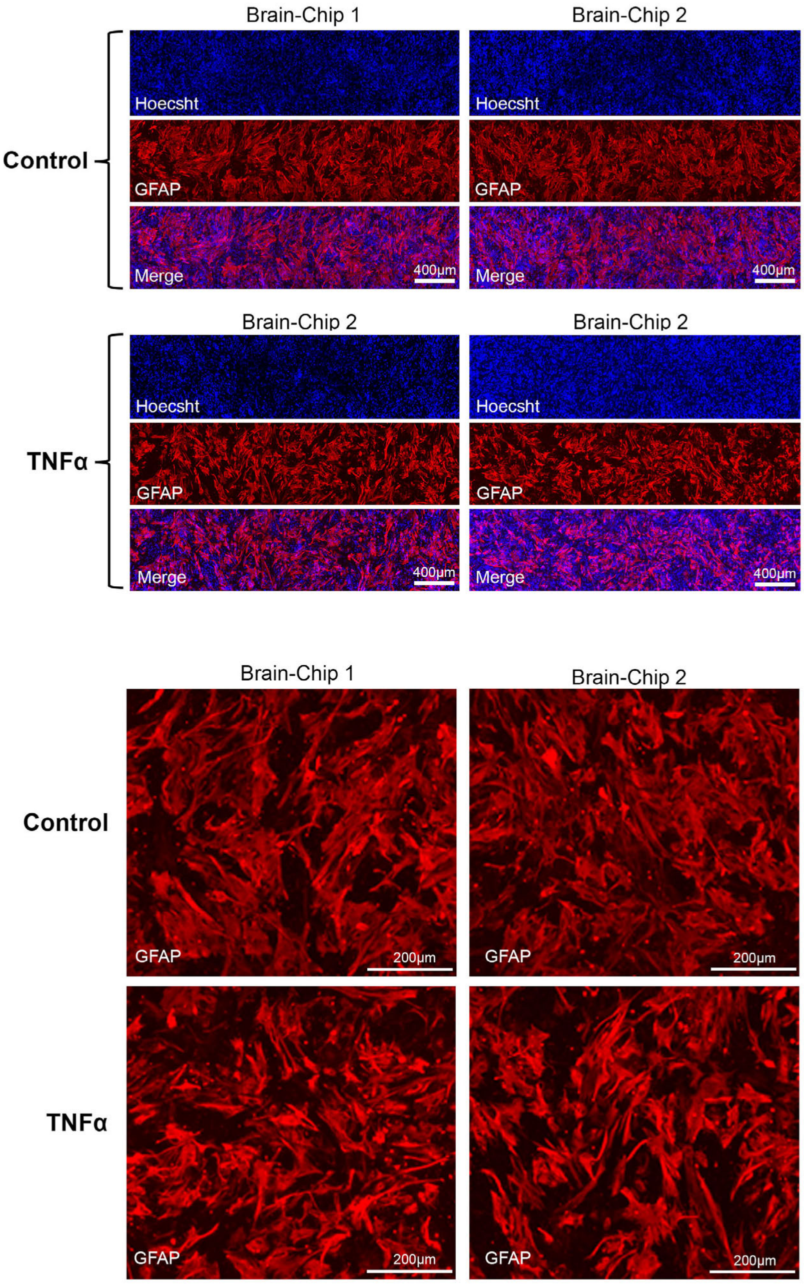

Figure S3. TNFα-induced morphological changes of astrocytes in the Brain-Chip. Confocal images (stack of z-series) of immunofluorescent staining against the astrocytic marker GFAP. Two chips per group were examined. Astrocytes transition from a polygonal to a more elongated shape upon exposure to TNFα. Supplementary data for Figure 2B.

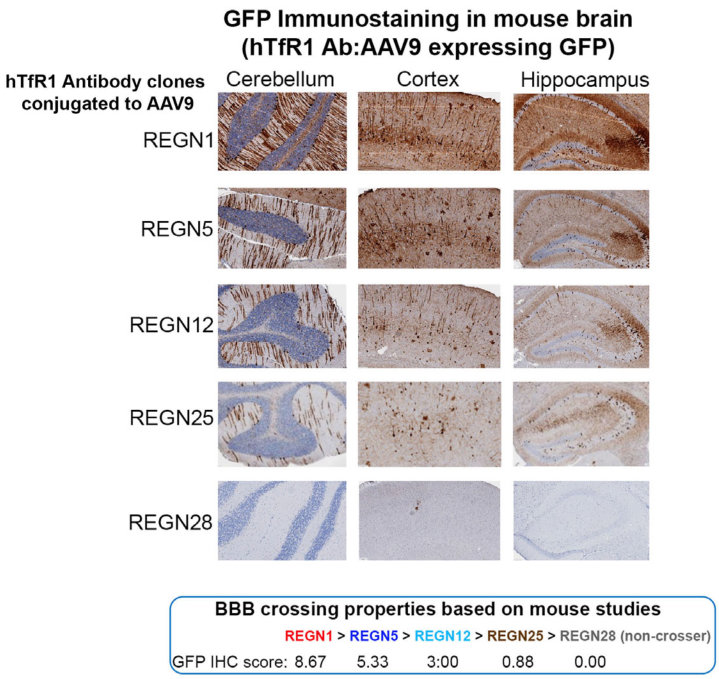

Figure S4. ***In vivo*** **tested hTFR1 antibody clones examined in the Brain-Chip for BBB crossing.** Representative images of GFP immunohistochemistry in different brain areas of hTfR1 knock-in mice injected i.v. with AAV9 conjugated with the indicated hTfR1 antibody clones. The crossing properties of the clones were determined based on the GFP signal in the mouse brain parenchyma and blinded quantification analysis. Score "zero" indicates no brain GFP signal and, therefore, no BBB crossing. The hTfR1antibody clones shown in the figure are part of a large iv vivo screening assay with thirty-two antibody clones. REGN1 was the best BBB crosser and REGN28 was among the clones that did not cross the BBB (no GFP signal). The GFP signal scoring for REGN1 and the rest of the antibody clones examined in the Brain-Chip is shown. Supplementary data for Figures 5 and 6.

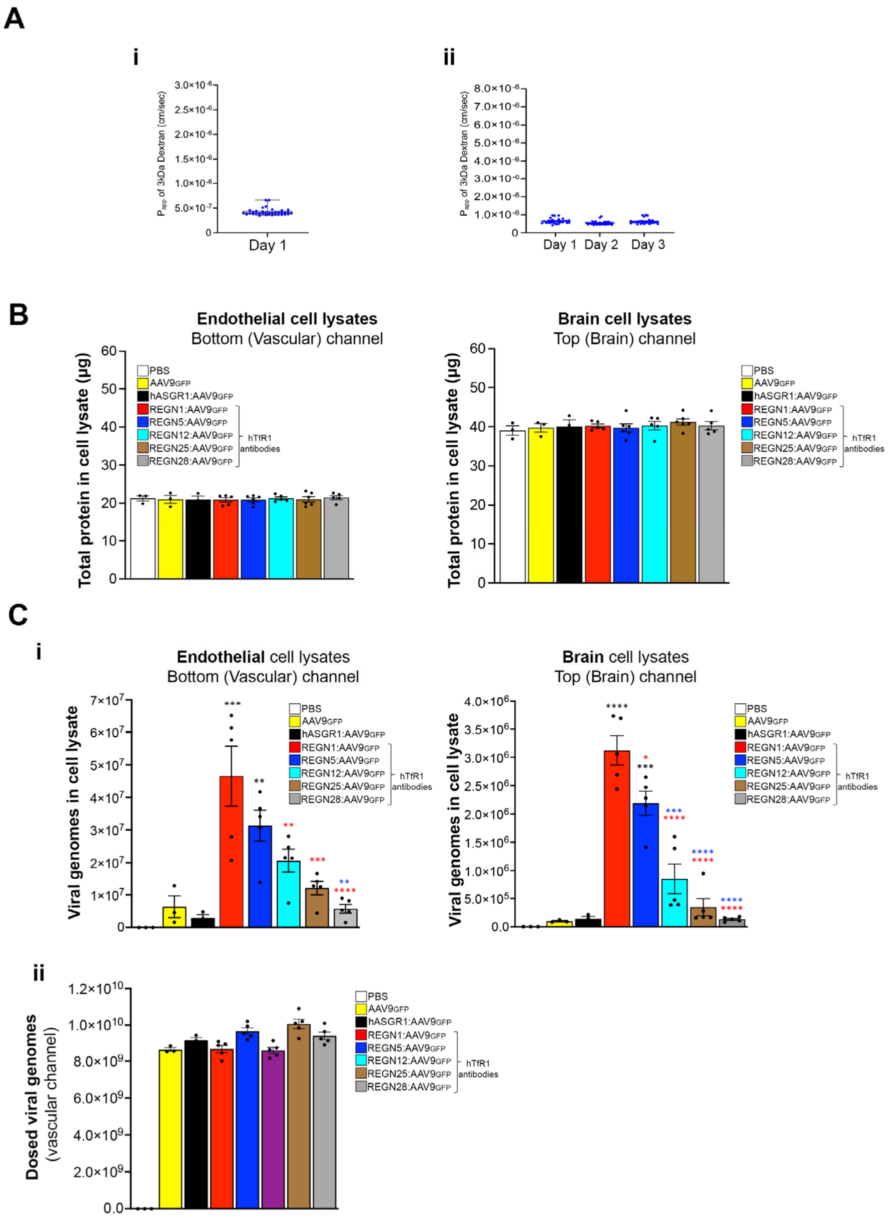

Figure S5. Additional experiments related to the testing of the resolution of the Brain-Chip to detect BBB crossing differences between hTfR1-specific antibodies. **(A)** Apparent permeability measurements in chips used for testing the hTfR1 antibodies as purified formats (Figure 5) (i) and conjugated to AAV9 (Figure 6) (ii). **(B)** Amount of total protein in endothelial cell and brain cell lysates used for the BBB crossing studies with hTfR1 antibody-conjugated viruses shown in Figure 5. No differences were observed between groups. **(C)** Quantification (Mean±SEM)of viral genomes in endothelial (i) and brain cells (ii) collected at the end of the AAV9 perfusion (day 3; Figure 6A). The differences between the groups were consistent with the GFP expression analysis experiment (Figure 6) and in agreement with the BBB crossing properties of the antibodies. (ii) Quantification of dosed viral genomes in the vascular media (measurements were performed prior to perfusion). hTfR1:AAV9: N=5 chips for each antibody clone; PBS, AAV9 and hASGR1:AAV9: N=3 chips per group. *$p < 0.05$, **$p < 0.01$, ***$p < 0.001$, ****$p < 0.0001$, one-way ANOVA with post hoc Tukey's test. Black asterisks: comparison with PBS, AAV9, hASGR1:AAV9. Red asterisks: comparison with REGN1. Blue asterisks: comparison with REGN5. Averaged data (Mean ± SEM) and individual chip values are shown (A-C).
